# Environmental footprint of a colonoscopy procedure: Life cycle assessment

**DOI:** 10.1055/a-2570-6599

**Published:** 2025-05-12

**Authors:** Paulina Lämmer, Dorien Oomkens, Tim Stobernack, Marjolijn Duijvestein

**Affiliations:** 1 International Business School Tuttlingen, Tuttlingen, Germany; 2 6034 Department of Gasteroenterology and Hepatology, Radboud University Medical Center, Nijmegen, Netherlands; 3 6034 Department of Intensive Care, Radboud University Medical Center, Nijmegen, Netherlands

**Keywords:** Endoscopy Lower GI Tract, Inflammatory bowel disease, Polyps / adenomas / ...

## Abstract

**Background and study aims:**

Gastroenterology is a specialty that has evolved rapidly over time, especially in terms of advancements in endoscopic procedures. However, these advancements also present challenges, given the substantial resource demands associated with endoscopy procedures. Numerous actions could be taken to develop a resilient healthcare system that consumes as few resources as possible, but recommendations are needed to prioritize which processes could be improved. We aimed to evaluate the environmental footprint of a colonoscopy procedure, and to identify the main contributing impact process categories.

**Methods:**

A single-center observational study was conducted at a Dutch university hospital. No clinical patient data were collected, but the colonoscopy procedure was studied. Data were collected during 13 colonoscopies. Life cycle assessment (LCA) was used to calculate environmental impact.

**Results:**

Damage to human health from one colonoscopy was 11.3·10 ^–5^ disability-adjusted life-years, equivalent to 1 hour. A single colonoscopy resulted in emission of 56.4 kg of CO _2_ -equivalent (CO _2_ eq), equal to driving a car for 255 km or 55 days of emissions for an average European household. Transportation of patients and staff (76.5%) and disposables (13.5%) were the greatest contributors to damage to human health.

**Conclusions:**

Among the 13 colonoscopies studied, the environmental impact was mainly attributable to transportation of patients and staff, and disposables. Therefore, raising awareness about the impact of transportation by car, and reducing resource consumption, particularly of disposable products, should be prioritized. Implementing alternatives to colonoscopy, such as intestinal ultrasound, could reduce the environmental footprint of the healthcare system.

## Introduction

The healthcare system, focused on enhancing overall health and well-being, paradoxically finds itself contributing to health challenges through its environmental footprint. Climate change stands as one of the most pressing global challenges of our time, with far-reaching implications for the environment, economies, and human health. The healthcare system emerges as a significant contributor to climate change, accounting for a significant 5% of the global net CO _2_ emissions, a proportion that is even larger in many industrialized countries 1 . As societies worldwide prioritize mitigating these challenges, various sectors, including healthcare, play a crucial role in reducing greenhouse gas emissions. Addressing climate change proactively not only enhances environmental sustainability but also strengthens healthcare systems' resilience, ensuring they can continue to provide effective care and protect public health.

Gastroenterology is a medical specialty that has evolved rapidly over time, especially in terms of advancements in endoscopic procedures. However, these advancements also present new challenges, given the substantial resource demands associated with endoscopy procedures. The Gastroenterology Department has the third highest amount of waste in the hospital 2 , and studies indicate that each endoscopic procedure generates between 1 and 3 kg of waste 3 . Due to this and other factors, endoscopy contributes to a considerable carbon footprint, with emissions ranging between 7.8 and 8.3 kg CO _2_ -equivalent (CO _2_ eq) per endoscopy, comparable to driving 36 km in a conventional car or 7 days of CO _2_ emissions from an average European household 4 .

Although the environmental impact of the healthcare system has often been overlooked, there is a growing shift in focus within the sector itself, government policies, and society towards addressing the system’s effect on the environment. A recent paper on climate change raises awareness about the significant environmental impact of the Gastroenterology Department, including endoscopic procedures, and underscores the urgent need and for sustainable initiatives, like “reduce, reuse, recycle” within the field 5 . There are numerous actions that could be taken to develop a resilient healthcare system that consumes as little resources as possible in the future, but recommendations are needed to prioritize which processes could be improved to reduce the environmental footprint.

This study specifically targeted the contribution of colonoscopy procedures to the environmental footprint. The impact and main contributing impact categories and processes were evaluated using a state-of-the-art life cycle assessment (LCA). Recommendations for environmental footprint reduction were developed based on these results.

## Methods

### Study design

A single-center observational study was conducted in the Radboud University Medical Center, a Dutch university hospital. No clinical patient data were collected, but colonoscopy was studied. Consumption of resources was determined by on-site visits and observations of the procedure. Environmental impact was determined by LCA. LCA is a method for calculating the environmental impact of a product or service, accounting for every phase from extraction of raw materials to disposal of waste.

### Data collection

**Fig. 1:**
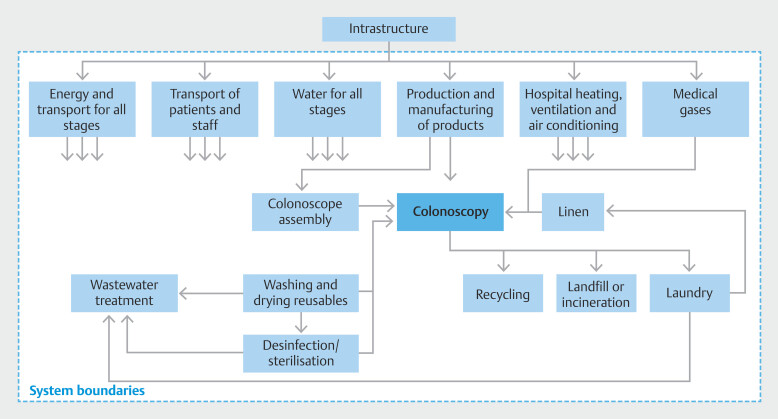
Boundaries of the LCA. For the different processes, the infrastructure required to enable production – such as energy, transportation, water, mining, gas and oil, biochemical, agricultural activities, and hospital and medical gases – was excluded from the LCA analysis. Transportation, production, manufacture, assembly, and reprocessing were included.

The functional unit was one diagnostic colonoscopy. Detailed data were collected during 13 diagnostic colonoscopies. Although no sample size calculation was performed, the number 13 was deemed sufficient to provide a reasonable data range, based on the standardized nature of colonoscopy procedures and previous comparable research 6 . During the colonoscopies, resources consumed in the colonoscopy process were analyzed. This included all used reusable and disposable materials and devices, water, chemicals, medication, energy consumption, and transportation of patients and staff. An overview of the analyzed inventory is shown in **Supplementary Material 1, Table 1, Table 2, Table 3, Table 4, Table 5, and Table 6.** The general hospital infrastructure was outside of the system boundaries. The boundaries of the LCA are shown in Fig. 1 .

Data were systematically retrieved between July 12, 2023 and the July 27, 2023 over a span of 6 separate days, distributed over 2 non-consecutive weeks. Colonoscopies were carried out by a total of 19 endoscopists, gastroenterologists as well as trainees, each operating with a varying team.

### Products

Life cycle data, i.e. information on weight, material, production, use, and waste processes, of the products used during colonoscopy were collected from an internal LCA database of the Radboud University Medical Center. If a product was not included in the LCA database, a new product was modelled by determining the weight and material composition; this method is described further in the paragraph below. Transport distances were based on the location of the production facility and calculated by Google Maps. Travel distances of staff and patients were also calculated using Google Maps. Staff were interviewed regarding their mode of transportation and patients were assumed to travel by car. Travel distances of staff were allocated based on the amount of time that the colonoscopy procedure occupied out of the total work day of the respective employee.

### Life cycle assessment methodology

LCA calculates the environmental impact based on all inputs and outputs to and from the environment during the raw material extraction, manufacturing, transport, use phase, and waste processing. Following ISO-14040/44 standards of the International Organization for Standardization (Geneva, Switzerland) 7 , we defined the following: The functional unit of the study was one diagnostic colonoscopy procedure. We included extraction of raw materials, production of plastics and manufacturing processes such as injection molding and extrusion, transport processes, and end-of-life processes such as incineration.

This method is comparable to previous LCA approaches performed by internationally recognized LCA researchers in the health care sector 6 8 9 . SimaPro 9 LCA software (PReʼ Sustainability, Amersfoort, the Netherlands) was used to model data. The ecoinvent database (version 3.9) was the primary source for global processes, including raw materials, manufacturing, transport, and waste. Complex products such as endoscopes and pharmaceuticals were modelled using these standard processes. Material composition of products was determined based on information from suppliers, material data sheets, expert opinion, and laboratory-scale weighing of components.

An inventory was created to measure the quantity of materials and energy consumed. The contribution of building energy was calculated based on an assumption made by the energy expert in our hospital, taking into account the ventilation system, surface area of the endoscopy room, and duration of the procedure. ReCiPe 2016 at midpoint and end-point level with the hierarchical perspective was used as the LCA method 10 . Midpoint analyses illustrate the environmental impact on 18 different environmental categories (eg, global warming, toxicity), whereas end-point analyses summarize the impact in a more aggregated category, e.g., damage to human health, expressed in disability-adjusted life-years (DALYs).

DALYs is a unit that describes the loss of healthy time in society, expressed in lost time for one person 11 . One DALY corresponds to 1 year, equivalent to 525,600 minutes 11 . Conversion of DALYs to time in our results was performed using this equivalence. The ecoinvent database served as a foundation for all models, whereas the internal Radboud University Medical Center LCA database contains medical products and services modelled using processes from ecoinvent.

## Results

Patients travelled an average 62 km round-trip to the hospital. During the 13 colonoscopy procedures, 44 products were used in total, 10 of which were reusable. An overview of the whole analyzed inventory is shown in **Supplementary Material 1, Tables 1, Table 2, Table 3, Table 4, Table 5, and Table 6** .

In total, damage to human health of one colonoscopy procedure was 11.3·10 ^–5^ DALYs, which is equivalent to approximately 1 hour. An estimated 11.0 million colonoscopies are performed yearly in the United States, resulting in the loss of 1,243 years of healthy lifetime or loss of 1 year of healthy lifetime for 1,243 people (1,243 DALYs) 12 .

The main contributing midpoint impact categories for damage to human health were global warming, fine particulate matter formation, and human carcinogenic toxicity. The global warming impact of one colonoscopy was 56.4 Kg CO _2_ eq, equivalent to driving a conventional car for 255 km or 55 days of CO _2_ emissions for an average European household. The midpoint impact categories are displayed in **Supplementary Material 2, Table 1** .

**Fig. 2:**
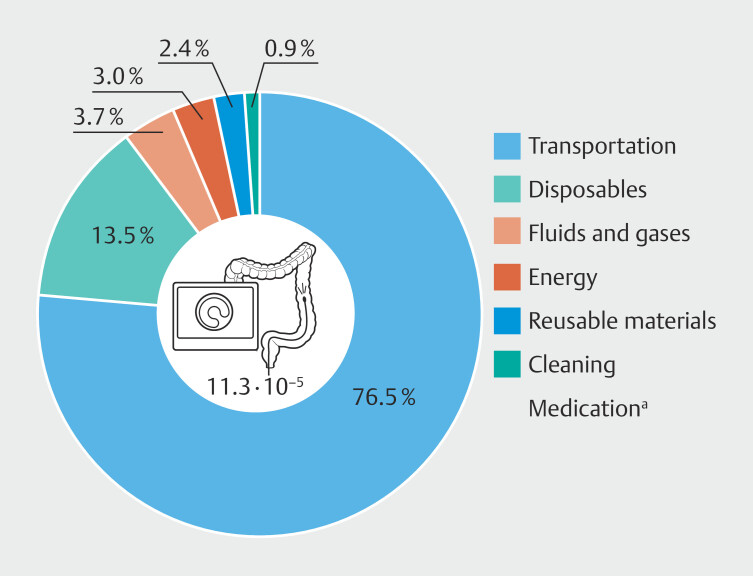
Percent contribution of different process categories on damage to human health. ^a^ = The contribution of medication (0.0069%) was so little that it could not be shown in the graph.

Processes impacting damage to human health were categorized into different groups: transportation of patients and staff, disposables, reusable materials, energy, fluids and gases, cleaning, and medication. Percentage impacts for these categories are displayed in Fig. 2 , with transportation of patients and staff having the greatest percentage impact on damage to human health.

When conducting impact assessment after excluding transportation of patients and staff from the analysis, total damage to human health for a complete colonoscopy procedure amounted to 2.7·10 ^–5^ DALYs, which is equal to 14.2 minutes.

The main contributing midpoint impact categories for damage to human health were fine particulate matter formation, global warming, and water consumption. One colonoscopy had a global warming impact of 14.2 Kg CO _2_ eq, equal to driving a conventional car for 65 km or 14 days of CO _2_ emissions for an average European household, and resulted in consumption of 137 L of water, equal to a 23-minute shower. Midpoint impact categories are shown in **Supplementary Material 2, Table 2** .

**Fig. 3:**
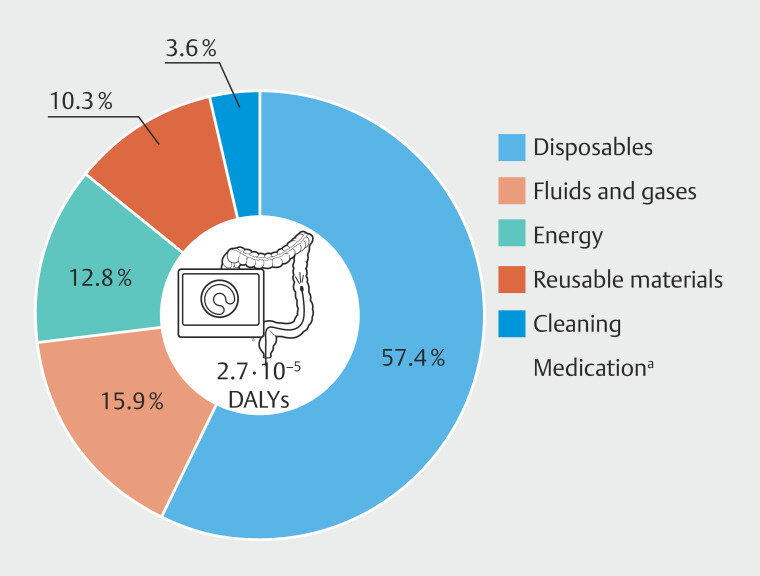
Percent contribution of different process categories on damage to human health, after excluding transportation of patients and staff. ^a^ = The contribution of medication (0.03%) was so little that it could not be shown in the graph.

Fig. 3 illustrates the percentage contribution to damage to human health of the different process categories involved in a colonoscopy procedure, after excluding transportation of patients and staff.

## Discussion

Transportation of patients and staff emerged as the greatest impact factor in our analysis, accounting for 76.5% of the total impact. Disposables were the second-largest contributor to damage to human health, accounting for 13.5% of total impact. A single diagnostic colonoscopy led to emission of 56.4 kg CO _2_ eq, equivalent to driving a conventional car for 255 km or 55 days of CO _2_ emissions for an average European household. Damage to human health per colonoscopy procedure was 11.3·10 ^–5^ DALYs, equivalent to 1 hour, meaning that for every patient who receives a colonoscopy, another person loses 1 hour of healthy lifetime. Without considering transportation, each colonoscopy leads to consumption of 137 L of water, which is equal to a 23-minute shower.

Research regarding the environmental impact of endoscopy procedures is scarce. Existing research has mainly focused on specific aspects of the procedure or specific facets of environmental impact, such as waste management. In an LCA conducted on commonly employed endoscopic instruments, researchers discovered that the instruments alone generated 67.74 kg CO _2_ eq over the course of 1 week of endoscopies 13 . They noted wide variability between manufacturers and they propose adoption of “green purchasing,” aiming to purchase similar quality instruments, but with less environmental impact. This could stimulate new competition in the market for instruments, which may lead to more sustainable production. Because this study reports CO _2_ eq emissions for 1 week of procedures without specifying the number of procedures performed during that time, direct comparison with our results is challenging.

Nevertheless, we consider the previous study highly important, particularly because we found that disposables were an important impact factor for damage to human health. A French retrospective study found that travel and medical equipment are major sources of the environmental impact of gastrointestinal endoscopy, which aligns with our results 14 . However, the carbon footprint that they found, 28.4 kg CO _2_ eq, was much lower than our result of 56.4 kg CO _2_ eq. This discrepancy might be attributable to differences in methodology and scope. Unlike our study, which consisted of an LCA, their results are based on retrospective data for 1 year. Furthermore, their study included all ambulatory endoscopies, whereas our study focused on a single diagnostic colonoscopy. Another study found that a single-use gastroscope had a 2.5 times higher carbon footprint than reusable gastroscopes 15 . This supports our findings about the significant environmental impact of disposables in endoscopy and highlights the need to transition to reusable alternatives. A study in which the yearly carbon emission of the gastrointestinal endoscopy unit was evaluated found that the carbon emission in 2022 was 62.72 tons CO _2_ eq 16 . The researchers excluded transportation from their analysis. In our analysis excluding transportation, we found a carbon emission of 14.2 kg CO _2_ eq for one colonoscopy, which equates to 33.6 tons CO _2_ eq in 2022 in our center, where 2369 colonoscopies were performed. The previous study examined all endoscopies, whereas our focus was solely on colonoscopies. Furthermore, carbon emissions from other endoscopic procedures have not yet been researched, making direct comparison of these results difficult.

We acknowledge several limitations. First, this study was conducted in a single hospital, specifically, a university hospital. This setting may not be representative of other healthcare facilities. University hospitals typically have more personnel present during procedures, leading to increased use of materials, such as gloves and surgical gowns, and more personnel present during a single colonoscopy. This can be seen in our results as well, because 13 colonoscopies were performed by 18 endoscopists. Some colonoscopies were performed by trainees, with supervision of a gastroenterologist. In addition, patients often travel longer distances to reach a university hospital compared with a regional hospital, which can affect the environmental impact related to transportation. Second, the number of colonoscopies analyzed in this study was relatively small, with only 13 procedures included. Because of our extensive data collection, it was not feasible to study much more than 13 procedures, but while colonoscopies are highly standardized, the limited sample size might not capture the full variability in practice and resource use across different settings and patient populations. Third, the travel data used in this study assumed that all patients travelled by car. While this assumption is likely accurate for many patients, it does not account for those who might use alternative modes of transportation. Finally, some data in the study are based on estimates. For instance, the exact reprocessing procedures for equipment could not be precisely documented, and usage of water and energy was not determined with exact measurements. These estimations introduce a degree of uncertainty into the analysis.

To our knowledge, this is the first study in which an LCA of a full colonoscopy procedure was performed. LCA was recently emphasized by Nordberg et al. 17 as the preferred method for quantification of the carbon footprint and other environmental effects of healthcare interventions. The LCA method provides a comprehensive evaluation of the environmental impacts associated with the entire life cycle of the procedure, enabling identification of key areas where resource use and emissions occur. This assessment allows for development of targeted strategies to mitigate these impacts, contributing to the broader goal of promoting sustainable clinical practices and reducing the carbon footprint of healthcare services. Another strength of this study is the standardized nature of the colonoscopy, which is based on well-established best practices and guidelines that are followed. This makes our results interpretable for other endoscopy units. Although the colonoscopy procedure itself is standardized, we studied multiple colonoscopy procedures to account for variations. Last, with this study we have identified several impact factors, making it possible to extrapolate recommendations to daily practice.

We found that transportation of patients and staff and disposable products accounted for the largest share of environmental impact of a diagnostic colonoscopy. Therefore, patients and staff should be made aware of the environmental footprint of travelling to the hospital by car, and should be encouraged to consider alternative modes of transportation. In addition, combining appointments to reduce visits can help minimize travel-related emissions. Reducing resource consumption, particularly disposable products, should also be a priority. During a colonoscopy procedure, a significant number of products are used. While not every product has a high individual impact, the cumulative effect across numerous procedures in a single hospital is substantial. When replacing a product or process with an alternative, the environmental impact should be carefully evaluated to ensure a positive overall outcome. In addition, we also emphasize the importance of recycling plastic, which contributes to approximately 10% of the generated waste in the endoscopy unit 18 . Recycling has already been demonstrated to be implementable through a brief training program for employees in the endoscopy unit in a previous study 18 . Table 1 summarizes specific recommendations for improving environmental impact in transport, disposables, energy consumption, and water consumption. Our full recommendations are presented in **Supplementary Material 3** , including our calculations for potential impact reductions in each category.

**Table:** **Table 1** Summary of specific recommendations.

Category	Main products/issues	Potential impact reductions
Transportation	Mainly car usage for patient and staff travel	Implement remote consultations; promote alternative travel modes such as bicycling or carpooling; combine appointments to reduce patient visits to the hospital on multiple days
Disposables	Disposable surgical gowns, endoscope covers, plastic cups	Replace with reusable options if feasible
Energy consumption	Reprocessing machines, heating, ventilation, and air conditioning systems	Optimize machine usage and minimize standby time
Water consumption	Reprocessing water usage	Upgrade machines for efficiency; reuse water where feasible

Another area of interest for improving environmental impact could be to explore more sustainable alternatives to colonoscopy. For instance, use of intestinal ultrasound to monitor inflammatory bowel disease has grown substantially and it could potentially be used as an alternative to colonoscopy. Intestinal ultrasound requires less plastic and paper than a colonoscopy, which reduces waste 19 . An LCA of diagnostic imaging in an Australian hospital found that one ultrasound results in emission of 0.5 kg CO _2_ eq 20 , significantly less than the emission of one colonoscopy we found, which was 14.2 kg CO _2_ eq. These CO _2_ emissions were both calculated without accounting for patient and staff transportation.

Given the limited literature on this topic, further research is warranted to validate our findings. Specifically, future research could concentrate more on reprocessing procedures, as well as gathering more detailed data on energy and water consumption for both the current and alternative processes. Moreover, conducting studies across multiple hospitals would help alleviate biases linked to hospital-specific variables. In addition, LCA of other endoscopic procedures would be of considerable interest, because that remains an unexplored area of research.

## Conclusions

This study identified hotspots in terms of environmental impact of a diagnostic colonoscopy, with transportation of patients and staff, and disposables accounting for the largest share. Exploring more sustainable alternative procedures, such as intestinal ultrasound, and promoting sustainable ways of transportation, resource use, and recycling can help reduce this footprint.

## Supplementary Material

Supplementary Material Zusatzmaterial

Supplementary Material Zusatzmaterial

Supplementary Material Zusatzmaterial
