# Supplementary material for: Environmental footprint of a colonoscopy procedure: Life cycle assessment
Source: Endosc Int Open. 2025 May 12;13:a25706599. doi: 10.1055/a-2570-6599 (PMC12080511; doi:10.1055/a-2570-6599)
Supplement: Supplementary file 1 — Supplementary Material [file 10-1055-a-2570-6599_25738011.pdf]

Supplementary 1 Analyzed inventory

Table 1 Medication.

Product	Amount	Range	Median
ALFentanyl	3 mL	0-6 mL	3 mL
Midazolam	3 mL	3 mL	3 mL
Propofol	38.5 mL	0-38.5 mL	0 mL
Remifentalyl	0.599 mL	0-0.599 mL	0 mL
K-Y Lubricating Jelly Sterile	10 g	10-20 g	10 mL

Table 2 Fluids and infusions.

Product	Amount	Range	Median
Sterile water		200-1000	300 L
CO_2	1.4 L/min	28-74.2 L	49 L
Tap water in cup for endoscope	250 mL	250-750 mL	250 mL
NaCl 0.9% infusion	1 L	0-1 L	0 L
NaCl 0.9% Posiflush	10 mL	10-20 mL	10 mL
Tap water for Infacol	250 mL	0-500 mL	250 mL
Simethicone (Infacol)	1 mL	0-2 mL	1 mL
Colex enema	133 mL	0-266 mL	0 mL
Formaldehyde	20 mL	0-120 mL	0 mL

CO_2, carbon dioxide; NaCL, sodium chloride.

Table 3 Electrical devices.

Product	Median
**Screens in the endoscopy room**	
Bed monitor	1
Monitor in endoscopy tower	1
Computer	1
**Lighting**	
Operating lights	4
Room light in between colonoscopies	4
Light in preparation room	1
**General room devices**	
Down flow ventilation unit	1
Label printer	1
**Endoscopy devices**	
Endoscope 1 colonoscope EC38-i10F2	1
Endoscope 2 colonoscope pediatric	0
Pentax medical endoscope tower EPK i7010	60 kWh
Endo stratus CO_2 insufflator	1
Water pump	1
Sunction pump	1
**Anesthesia devices**	
GE dinamap procare 300	1
Anesthesia carestation GE Avance CS2	0
Pulse oximeter	0
Blood pressure meter	0

CO_2, carbon dioxide.

Thieme

Table 4 Reusable materials.

Product	Median
**Patient materials**	
Sheet under patient	1
Sheet pillow	1
Cover patient	1
Blood pressure band	1
Pulse oximeter	1
**Endoscopy**	
Metal bowl for syringes	1
Plastic box for endoscope transportation	1
**Clothes for personnel**	
Trousers	4
Shirt	4
White coat	4

Table 5 Disposable materials.

Product	Median
**Medication administration**	
Syringe Plastipak 3 mL	2
Blunt fill needle	2
Sedation	
Nasal cannula for oxygen	1
Intravenous line	1
Sterile sticker Tegaderm for IV	1
NaCl 0.9% infusion liquid 1000-mL bag	0
EKG stickers	0
**Endoscopy**	
Syringe Plastipak 50mL	1
Water cup for endoscope cleaning	1
Water cup infacol	0
Water cup colex	0
Suction bag	1
Hoses for suction bag	1
Hoses for water/flushing endoscope	1
Bottle of sterile water 1L	0.3
Captivator 10 mm	0
Biopsy forceps 2.8mm	0
Rectal tube	0
Polyp trap	0
Plastic cover endoscope box with label	1
Plastic over endoscope box	1
Plastic sheet under patient	1
Biopsy channel cap, rubber, 30 g	1
Cap for endoscope distal tip	1
Tissue for jelly	1
Container biopsy formaldehyde 3.8%	0
**General disposables**	
Coat	3
Face mask	0
Gloves	8
Plastic glasses	1
Cleaning	
Hand disinfectant	5
General disinfectant	1
Cleaning tissues	5
Paper towels	5

IV, intravenous; NaCL, sodium chloride.

Table 6 Cleaning of bed after the colonoscopy.*

Product	Median
Gloves	0.1
Plastic coat	0.05
Plastic polyester single use tissue	0.15
Duster	0.05
Microfiber tissue	0.15
Disinfection Terralin protect	500 mL

*Four patients per day, cleaning once per week

Endosc Int Open | DOI 10.1055/a-2570-6599 | © The Author(s).
