# Supplementary material for: Environmental footprint of a colonoscopy procedure: Life cycle assessment
Source: Endosc Int Open. 2025 May 12;13:a25706599. doi: 10.1055/a-2570-6599 (PMC12080511; doi:10.1055/a-2570-6599)
Supplement: Supplementary file 2 — Supplementary Material [file 10-1055-a-2570-6599_25738013.pdf]

Supplementary 2 Additional results.

Table 1 Main contributing midpoint impact categories.

Impact category	Unit	One colonoscopy	Yearly in the United States*
Global warming	Kg CO_2eq	56.4	620,400,000
Fine particulate matter formation	Kg $PM_{2.5}$eq	0.054	594,000
Human carcinogenic toxicity	Kg 1.4-DCB	4.22	46,420,000

*Estimated 11.0 million colonoscopies yearly in the United States.

CO_2, carbon dioxide, DCB, dichlorobenzene; Eq, equivalent; $PM_{2.5}$, particulate matter 2.5.

Fine particulate matter formation is an air pollutant that poses health risks when its concentration in the air exceeds 35 µg/m^3.

Human carcinogenic toxicity is the potential of substances or exposures to cause cancer in humans by damaging DNA, promoting abnormal cell growth, or disrupting normal biological functions.

Endosc Int Open | DOI 10.1055/a-2570-6599 | © The Author(s).

Table 2 Main contributing midpoint impact categories after excluding transportation.

Impact category	Unit	One colonoscopy	Yearly in the United States*
Fine particulate matter formation	Kg $PM_{2.5}$ eq	0.0126	138,000
Global warming	Kg CO_2eq	14.2	156,200,000
Water consumption	L	137	1,507,000,000

*Estimated 11.0 million colonoscopies yearly in the United States.
DCB, dichlorobenzene; Eq, equivalent; $PM_{2.5}$ = particulate matter 2.5.
Fine particulate matter is an air pollutant that poses health risks when its concentration in the air exceeds 35 µg/m^3.
