# Supplementary material for: Environmental footprint of a colonoscopy procedure: Life cycle assessment
Source: Endosc Int Open. 2025 May 12;13:a25706599. doi: 10.1055/a-2570-6599 (PMC12080511; doi:10.1055/a-2570-6599)
Supplement: Supplementary file 3 — Supplementary Material [file 10-1055-a-2570-6599_25738014.pdf]

Supplementary 3 Recommendations

Following is a discussion of possible measures and actions to improve the overall environmental impact by reducing the impact of the hotspot processes, with figures for potential reduction in CO_2eq. Furthermore, the potential financial savings related to these measures are discussed.

Transportation

The transportation of passengers emerges as the predominant contributor, thus offering significant potential to reduce the footprint. The high impact of transportation can be largely attributed to use of cars with combustion engines. These are mainly used by patients and physicians for driving rather long distances. Using a car is the most convenient option for longer distances. Although it is challenging, there are some actions that can be implemented to change and improve the travel pattern. First, appointments for patients in the hospital can be reduced as much as possible. This means that besides the colonoscopy examination, all other consultations should preferably be carried out on the phone or in an online appointment. Physicians should actively engage in scheduling remote appointments with their patients and also explain the reason in order to create awareness of and acceptability by patients. To change travel behavior of employees, a combination of incentives and offers could be implemented. To incentivize use of alternative modes of travel, an app-driven challenge to collect points for using alternative modes of transportation and that can be used to obtain something such as vouchers has been successfully tested in other hospitals (Luisenhospital Aachen 2023). To make it as attractive as possible to use a bicycle, maintenance free of charge, storage spaces, charging stations, and bike-leasing models could be implemented.

Disposables

There are some disposable products that have a noteworthy contribution to the overall impact. The main contributing products are disposable surgical gowns for reprocessing and covers for endoscope transportation boxes. Some other products have a rather small contribution but are easy to replace with reusable alternatives and thus are discussed as follows:

1. *Endoscope transportation box cover*

Covers for endoscope transportation boxes emerge as significant contributors to the overall impact and could be replaced with reusable covers. These covers could then be cleaned as is done with boxes used for disinfecting cleaning tissues. Replacement of disposable covers would lead to a reduction in global warming impact per piece of 1.13 kg CO_2eq and a net reduction of approximately 1.1 kg CO_2eq (taking into account the wipe cleaning tissue that is also needed). This totals up to 2,600 kg CO_2eq per year that can be saved for all colonoscopies performed at Radboudumc (2369 in year 2022). Assuming that other endoscopic procedures have the same transportation boxes, the annual net savings for the Gastroenterology Department (6948 endoscopic examinations in 2022) by replacing the disposable covers would amount to 7,600 kg CO_2eq. It should be mentioned that the reusable cover does not fit currently used boxes. Thus both would need to be replaced. Thus, from an environmental and also economical perspective, it would be preferable to change to boxes with reusable covers at the end of the boxes' usable life.

2. *Disposable gowns for reprocessing staff*

Surgical gowns, which are used by reprocessing staff, have a high contribution to the overall impact, based on the assumption that they are changed for every endoscope, as is recommended by most guidelines. Global warming impact for each endoscope accounts for 2.92 kg CO_2eq, totaling up to nearly 7,000 kg CO_2eq for colonoscopies performed annually at Radboudumc. Taking into account washing of reusable gowns and assuming a similar impact as washing staff linen clothes because they do not need to be sterile, a possible improvement would be to use reusable surgical gowns. Net reduction in global warming impact would amount to 2.1 kg CO_2eq per endoscopy and almost 5,000 kg CO_2eq for the colonoscopies performed annually at Radboudumc. One downside argument that should be taken into account is the high number of reusable surgical gowns that would need to be stocked, considering their frequent replacement for each endoscope and the high number of endoscopes that are reprocessed per day. The cost for one disposable gown is approximately €1.90. Conversely, the expenditure for a reusable gown is estimated at roughly €20 and would thus be amortized in a short time of reprocessing 11 endoscopes. Annual monetary savings would amount to 4,500€ for colonoscopies performed at Radboudumc. Costs for the laundering process per piece are difficult to estimate. Taking into account possible savings in terms of initial costs and environmental footprint, the change to reusable gowns would have an overall positive benefit.

3. Plastic water cup

The water cup for the endoscope bedside cleaning could be replaced by a metal cup that can be reprocessed in the washer-disinfector machine. This would require a washer-disinfector machine with a basket in which the endoscopes and the metal cup can be placed. Reprocessing of the cup would not have any additional impact because the washer-disinfector machine runs irrespective of the cup for disinfecting the endoscope. In comparison to other processes, the impact of one cup is rather small, because the cup is relatively small and lightweight. Nevertheless, replacement of it with a reusable alternative would lead to savings of 0.17 CO_2eq per colonoscopy and an annual reduction in global warming impact of 400 kg CO_2eq for all colonoscopies performed at Radboud. Assuming that the same cups are used for other endoscopic processes, the amount would be even higher, for instance 1.200 kg CO_2eq that could be saved only for the Gastroenterology Department.

4. Disposable bed liners

Disposable bed liners that are used for colonoscopy have a global warming impact of 0.17 kg CO_2eq per colonoscopy. The amount is rather small in comparison with other processes. There are reusable alternatives that could be used, but they would not significantly improve the impact because they would need to be washed. The laundering process would offset the impact of savings from the disposable product. In addition, from a practicality and usability point of view, disposable bed liners are more user-friendly and integratable into the process of the colonoscopy.

5. Distal tip protector

The distal tip protector that is used to avoid damages to the sensitive distal tip of the endoscope is currently a disposable sterile product. Global warming impact of 0.07 kg CO_2eq per piece is relatively small. However, the accumulated impact amounts to 165 kg CO_2eq for the colonoscopies performed annually at Radboud and 486 kg CO_2eq for the Gastroenterology Department (assuming usage of the distal tip protector for any kind of flexible endoscope). The disposable product could be replaced by reusable solutions that can be reprocessed together with the endoscope, and thus, not have any additional impact through reprocessing.

Energy consumption

The main energy consumption can be attributed to machines used in the reprocessing unit, namely the washer-disinfector and drying cabinet, as well as the

HVAC system in the treatment room. Reprocessing devices have a global warming impact of 0.9 kg CO_2eq per colonoscope. One solution to reduce energy consumption per endoscope would be to have washer-disinfector machines that can reprocess multiple endoscopes simultaneously. Especially for a hospital with a large number of endoscopes per day, this would be also be a possible improvement in terms of process optimization. Furthermore, the machines could be switched off during the night instead of leaving them in standby mode. The exact reduction of global warming impact depends on the energy consumption of washer-disinfector machines that would be used alternatively. A rough estimation can be done assuming an energy saving of 30% per endoscope if three or four endoscopes are reprocessed at the same time. Switching off the machines during the night instead of leaving them in standby mode would only insignificantly reduce the impact. Cumulatively, these measures would lead to a reduction on global warming impact of 0.04 kg CO_2eq per endoscope and of almost 95 kg CO_2eq annually for all colonoscopies performed at Radboudumc. It should be mentioned that replacement of reprocessing devices is related to large investments and structural and organizational efforts. Therefore, the recommended action could be taken into account as soon as the devices reach the end of their usable life and will be replaced.

Water consumption

For the analysis performed including transportation, the main hotspot for water consumption lies within the transportation process. By investigating the numbers excluding the transportation from the analysis, a high amount of water is used in reprocessing. The precleaning procedure and cleaning and disinfection in the washer-disinfector machine consumes almost 80 L of water. Similar to the argument for energy consumption of the washer-disinfectors, machines with the ability to clean more than one endoscope at the same time would consume less water per endoscope. The same assumption of 30% water reduction can be applied, leading to an annual reduction of water consumption of 28,000 L for all colonoscopies that are carried out annually at Radboudumc. Furthermore, precleaning requires a large amount of water because the basin is newly filled after each endoscope. Separating the dirt that is brushed out of the channels during the precleaning process would probably allow use of the water several times. The outer surface is already wiped

cleaned and the main contamination is in the inner channels. Actions that are taken to reduce water consumption have the additional positive effect of also reducong consumption of detergents and disinfectant chemicals.

Conclusions about recommendations

As the analysis has shown, disposable products have the highest share of overall impact, excluding transportation. There are a large number of products used during colonoscopy. Not all of them have high contributions as individual products. However, the sum over many procedures that are conducted in one hospital always leads to noteworthy impacts. For this reason, reducing resource consumption, and in particular, disposable products, should always be aimed for. A similar argument holds for use of electricity, fluids, and gases. If replacing one product or process requires an alternative product or process, the impact should be compared because it may not have a positive net outcome, as the example of the disposable bed liners has shown.

For the majority of processes in which disposable products can be substituted with reusable alternatives, one can assume not only a reduction in emissions but also potential monetary savings, as shown by the example of the disposable gowns for reprocessing. For most of the products, obtaining precise cost data and subsequently calculating costs of the alternatives was not possible. Typically, the initial investment in a reusable product is amortized in a short period of time. All measures related to energy or water consumption reduction would lead to monetary savings and are even more important in the context of increasing energy costs.
